# Toxicological evaluation of a polyherbal formulation on testicular function and gonadal histomorphology in exposed Wistar rats

**DOI:** 10.37796/2211-8039.1404

**Published:** 2023-06-01

**Authors:** Godswill James Udom, Uduak Peter Ise, Omoirri Moses Aziakpono, Ayodeji Aturamu, Mba Ogbonnaya, Israel Kevin Umana, Jude Efiom Okokon

**Affiliations:** a Department of Pharmacology and Toxicology, Faculty of Pharmacy, Federal University Oye Ekiti, PMB 373, Oye-Ekiti, Nigeria; b Department of Pharmacology and Toxicology, Faculty of Pharmacy, Bingham University, Karu, Nigeria; c Department of Human Physiology, College of Medicine and Health Sciences, Afe Babalola University, Ado-Ekiti, Nigeria; d Department of Pharmacology and Toxicology, Faculty of Pharmaceutical Sciences, Nnamdi Azikiwe University, Akwa, Nigeria; e Department of Pharmacology and Toxicology, Faculty of Pharmacy, Madonna University, Elele, Nigeria; f Department of Pharmacology and Toxicology, Faculty of Pharmacy, University of Uyo, Nigeria

**Keywords:** ***Keywords:*** Testicular function, Sperm morphology, Herbal remedy, Gonads, Toxicity

## Abstract

**Introduction:**

Dr Iguedo Goko Cleanser® is a herbal formulation (HF) widely marketed in southern Nigeria and purported to be very efficacious for the management of various diseases including giardiasis, toilet infections, hypertension, diabetes, ulcer, impotence, low libido, low sperm count amongst others. Medicinal plants reportedly produce an array of adverse reactions capable of inducing harmful conditions, including death.

**Aim:**

This study evaluated the subchronic toxicity concern of HF on testicular function and gonadal histoarchitecture in Wistar rats.

**Methods:**

Thirty Wistar rats of both sexes were randomly divided into six groups (5/group) and were orally administered HF for 60 days. The control groups received 5 mL/kg of distilled water; the treatment groups were administered 476.24 and 158.75 mg/kg body weight of HF each for both male and female rats. Using standard procedures, semen analysis was done for all male rats. Animals were anaesthetised and sacrificed on the 62nd day; the gonads were eviscerated, weighed and fixed in 10% buffered formalin for histopathological examinations.

**Results:**

Significant (p < 0.05) increase in sperm count relative to control as well as spermatotoxic effects were observed in male rats. Histologically, the ovary presented some degrees of pathologies: cloggy appearing ovarian cortex with a display of a tumour-like cortical area, scantily displayed primordial follicles, haemorrhagic blood vessels, atretic secondary follicle, and eroding granulosa cells amongst others. Testicular histopathology showed abnormal seminiferous tubules’ histoarchitecture, degenerated spermatids, distorted spermatogenic cells’ orientation, and displaced spermatids into the luminal space.

**Conclusion:**

Herbal drugs are usually regarded to be completely safe due to their natural sources, however, this study discovered exposure-related toxic effects of Dr Iguedo Goko Cleanser® on testicular function and gonadal histomorphology. The findings recommend extreme caution with chronic use and avoidance whenever possible.

## 1. Introduction

The gonads are very critical to reproduction both in mammals and other organisms. The gonads are specialized organs that bear the germ cells. In organisms that are sexually differentiated, the gonads are gender-based, i.e., males have testes and females have ovaries. Typically, especially in organisms needing post-coitus *in vivo* fertilization, the gonads are connected to the primary sex organs (penis and vagina), release the gametes as well as involved in the production and regulation of reproductive hormones. These gonadal functions if and when altered either by drugs, chemicals or existing disease conditions may result in infertility. In the human context, the latter refers to the difficulty of married ones to conceive after one year of adequate coitus without the use of contraception and is a major home-shattering and/or grief striking condition. Globally, this condition represents a major health and social problem [1].

Infertility is often multifactorial with 40% male, 40% female, 15% combined and 5% unexplained factors respectively [1].

Dr Iguedo Goko Cleanser® is a polyherbal formulation licensed and approved for circulation by a national regulatory body under the registration number A7-0804L. It is popularly promoted among native Nigerians in the south-west, south-east and south–south regions as a potent remedy for an array of ailments such as giardiasis, toilet infections, hypertension, diabetes, ulcer, cancer, impotence, low libido, low sperm count amongst others [2]. It comprises five medicinal plants: bitter leaf, garlic, ginger, sugarcane and pigeon pea [3]. The use of herbal remedies has increased tremendously in the Sub-Saharan African region and is chiefly due to their perceived efficacy and safety. Following the claim of a 100% safety margin, herbal formulations are widely distributed in this region without any mandatory regulation. The major issue bothering these quasidrug formulations is the issue of standardization and quality assurance. Therefore, a greater number of these preparations may be contaminated with heavy metals, polycyclic aromatic hydrocarbons (PAHs) as well as other chemicals and/or may contain impurities that endanger human physiology. As a result, exposure to these commonplace items is a major public health concern. It is important to note that the combination of many medicinal herbs as is the case with many herbal remedies distributed in Nigeria could trigger a cascade of interactions with probable deleterious health outcomes. Numerous studies have associated herbal remedies with cases of poisoning [4–6]. Presently, there is a dearth of information on the toxicological evaluation of numerous herbal remedies distributed in the Niger–delta region of Nigeria both in animal and human studies. Therefore, this research was designed to determine the 60-day exposure-associated outcomes of Dr Iguedo Goko Cleanser® on testicular function parameters and histomorphology of the gonads using a rat model. It is thought that such findings will provide a rationale for the safety of this product vis-à-vis help protect public health against exposure-associated adverse health effects.

## 2. Methods

### 2.1. Stock solution

The polyherbal formulation was purchased from a major distributor in the Uyo metropolis, Nigeria, and the stock concentration was determined as earlier reported by Udom et al. [7].

### 2.2. Experimental animals

Thirty Wistar rats of both sexes between the weight range of 120 and 160 g were purchased from and kept in sterilized polypropylene cages with sterile paddy husk as bedding at the Animal Unit, Department of Pharmacology and Toxicology, Faculty of Pharmacy, University of Uyo, Nigeria. Before the experiment, the animals were quarantined and allowed to adjust to their immediate environment (laboratory) for at least one week, during which they were allowed free access to food and water at will. The animals were handled in line with the ARRIVE guidelines. To minimize potential differences that may arise during the experiment, experimental animals within the same weight range were caged together and marked with picric acid either as head, back, tail, plain, or right ear for easy identification during drug administration. Based on their positions on the rack, the six cages used were numbered accordingly.

### 2.3. Experimental design

Thirty healthy adult Wistar rats of both sexes (15 each; 120–160 g) were divided at random into six groups consisting of five rats per group (3 male groups and 3 female groups) and exposed to the herbal formulation as presented in Table 1. Random numbers were generated using Microsoft Excel (standard = RAND (o) function). The small sample size was selected following the 3Rs (reduction, refining and replacement) principle as regards the use of animals in research. The doses were selected using the LD50 (30 and 10%) as previously determined and reported [3] and were administered daily between 8 and 9 am using oral gavage for 60 days [8]. The experimental animals in the various groups were closely monitored for changes in behaviour, drinking and eating patterns. Also observed were changes in body weight and general morphology (eye, fur, stool and urine colourations). At the expiration of the test duration, all experimental animals were euthanized and sacrificed under carbon dioxide (Sigma, USA) anaesthesia. The testes and ovaries were eviscerated from each euthanized animal for histopathological assessment.

### 2.4. Semen analysis

From each euthanized male rat, the seminal vesicles and caudae epididymis were eviscerated and dropped gently into a beaker containing 1 mL of physiological saline and excised so that the sperm can freely swim out into the solution. The beaker was kept steady until the semen was completely liquefied. The solution was thoroughly mixed and 5 μL was collected with a micropipette and delivered onto a glass slide and covered with a 22 × 22 mm cover slip. The spermatozoa were assessed for motility and morphology using a high-powered microscope at × 400 magnification. Motility estimation is carried out at room temperature between 24 and 28 °C and recorded in percentage and classified as actively motile, sluggish and non–motile sperm cells [9–12]. Spermatotoxic effects were determined by close observation and count of abnormalities in sperm cell morphology (defects in the head, midpiece and tail) [9,10].

For sperm count and concentration, 50 μL spermatozoa were diluted in 950 μL of semen diluent. The solution was thoroughly mixed and a few drops were introduced into a haemocytometer Neubauer’s counting chamber, and the spermatozoa were assessed for count using a high-powered microscope at × 100 [13]. The Average number of cells and cell concentration were calculated using the formula,


Equation 1 $$\text{Average number of cells}=\frac{\text{Sum of cells in each square}}{\text{Number of squares}}$$

### 2.5. Histopathological assessment

From each carbon dioxide euthanized and sacrificed rat, the testes and ovaries were eviscerated, blotted with tissue paper, weighed fresh, sectioned and fixed in 10% buffered formalin for histopathological examination. The fixed sections were first dehydrated with alcohol, then cleared with xylene, infiltrated, mounted with paraffin wax, sectioned, rehydrated, stained with haematoxylin and eosin, and finally mounted with coverslips for the histopathological assessment under a light microscope at a magnification of × 100 [14]. To reduce bias, the pathologist was not informed of the treatment regimens administered to the experimental animals [15].

### 2.6. Statistical analysis

Statistical analysis was done using SPSS version 17 (SPSS Inc., USA), and statistical significance between the groups was analysed via one-way analysis of variance (ANOVA) followed by LSD as a post-hoc test. The results were presented as Mean ± S.E.M. The statistical significance was set at p < 0.05.

### 2.7. Ethical consideration

This study was approved by the Experimental Ethics Committee on Animal Use, Faculty of Pharmacy, University of Uyo, Nigeria and was conducted in strict compliance to ARRIVE guidelines and the Guide for the Care and Use of Laboratory Animals [16].

## 3. Results

### 3.1. Semen analysis

**Fig. 1:**
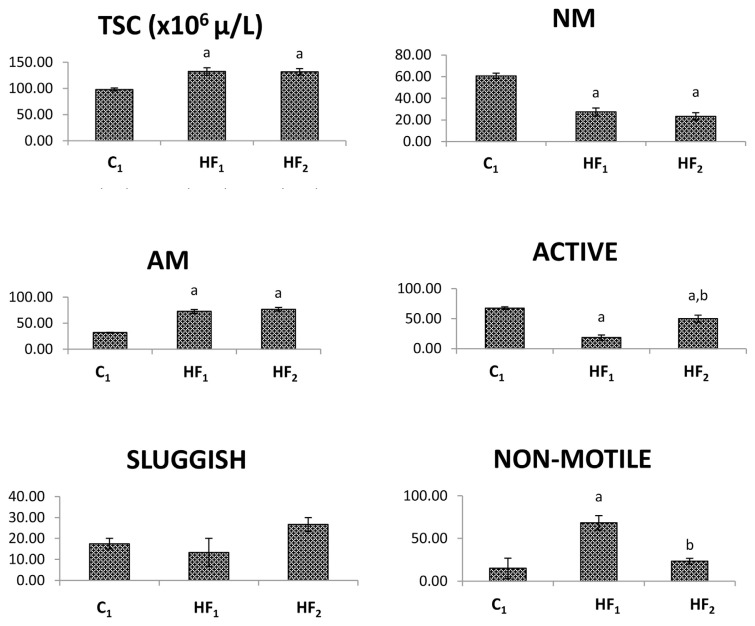
Semen analysis of Wistar rats exposed to the herbal formulation. Data presented as Mean ± Standard Error of Mean (SEM). Compared means are considered statistically significant at p < 0.05; a = significantly different when compared to C_1_ (control); b = significantly different when compared to HF_1_ (high dosed males); HF_2_ = low dosed males; TSC = total sperm count; NM = normal morphology; AM = abnormal morphology; n = 5.

At the doses tested, HF caused a significant (p < 0.05) increase in sperm count compared to the control. Also, a significant decrease in normal morphology and a corresponding increase in abnormal sperm morphology were recorded in comparison with the control at all doses tested (Fig. 1). Exposed male rats had significantly reduced actively motile sperm relative to the control. However, male rats exposed to low doses of the herbal formulation had increased actively motile sperm relative to those exposed to high doses. Non-motile spermatozoa were respectively elevated and reduced in male rats exposed to high and low doses of the herbal drug compared to the control (Fig. 1).

### 3.2. Histopathological assessment

**Fig. 2:**
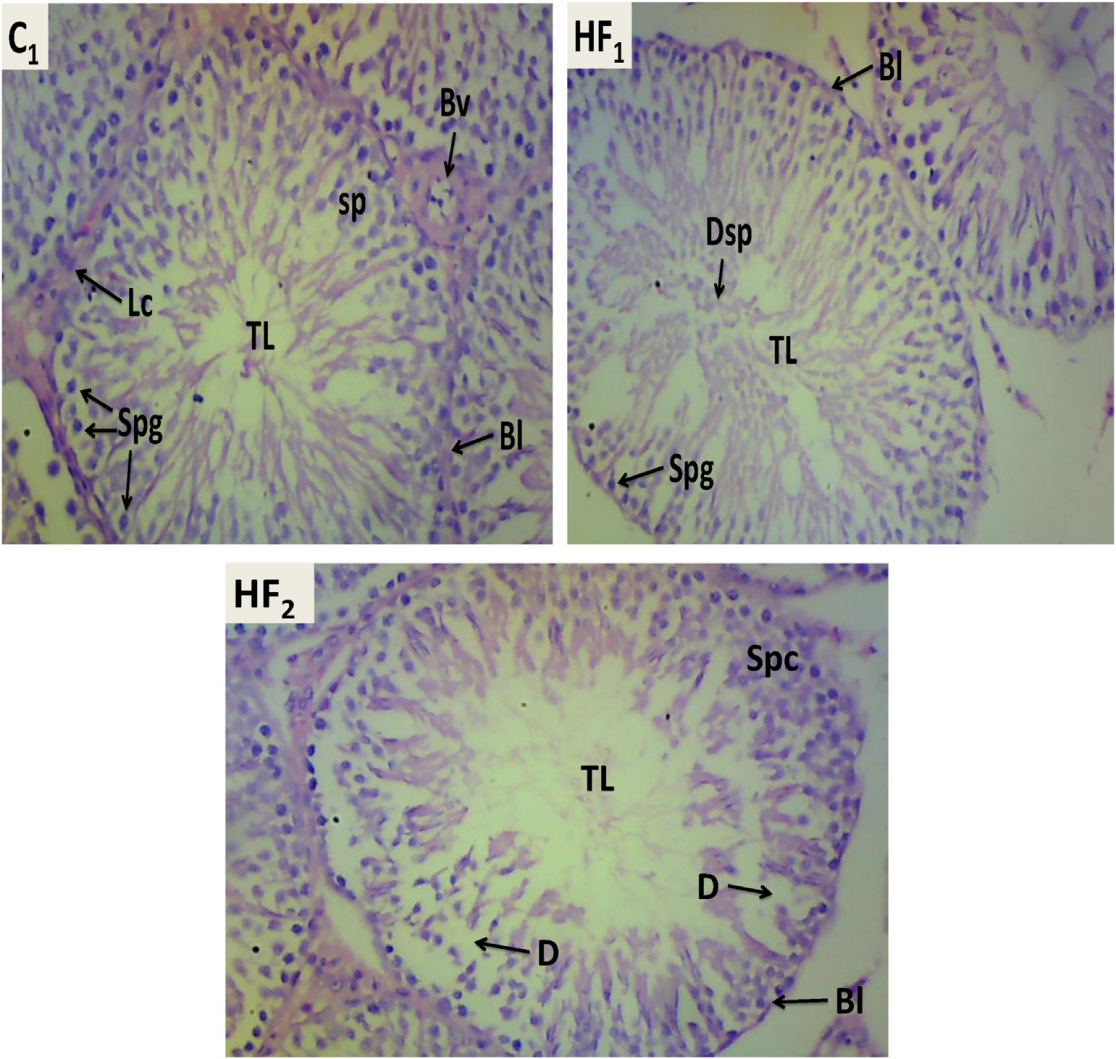
Typical transverse sections of testis from control (C_1_) and high and low doses exposed male rats (HF_1_ and HF_2_) respectively showed normal histoarchitecture of the seminiferous tubule, well-lined basement layer (Bl), normal orientation of the spermatogenic cells (Spc), presence of blood vessels (Bv) and Leydig cells (Lc) within the interstitial space; abnormal histoarchitecture of the seminiferous tubule, well-lined basement layer, mildly distorted orientation of the spermatogenic cells, displaced spermatids (Dsp) into the luminal space, presence of blood vessels and Leydig cells within the interstitial space; mild distortion in the histo-arrangement of the seminiferous tubule cells, well-lined basement layer, normal orientation of the spermatogenic cells, and areas of degeneration (D) of spermatids ×100 magnification.

**Fig. 3:**
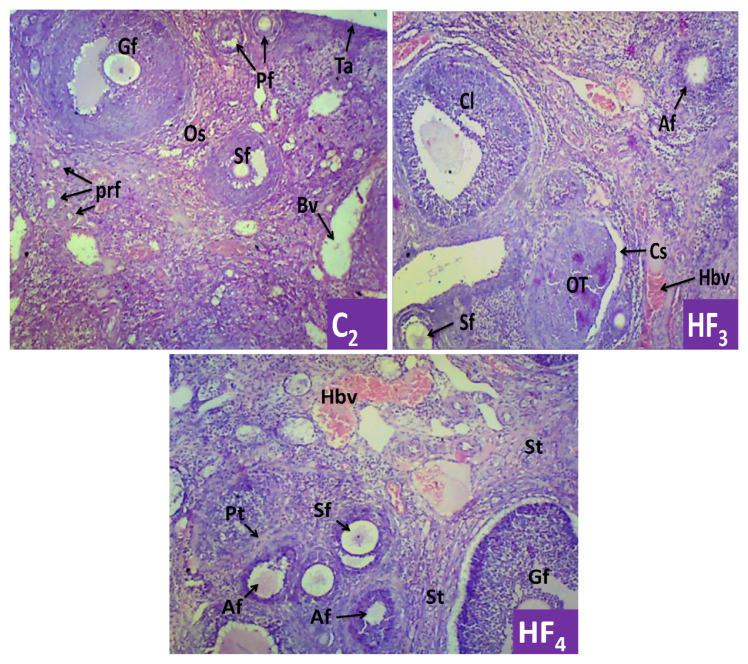
Typical ovary sections from control (C2) and high and low doses exposed female rats (HF_3_ and HF_4_) respectively showed normal histoarchitecture of the ovarian cortex with a display of different levels of follicular developments, well lined ovarian stroma (Os) with normal orientation of the connective tissues, and presence of widely space primordial follicle (Pf) and blood vessels (Bv); cloggy ovarian cortex with display of a tumour-like cortical area with a cystic space, scanty primordial follicle (prf), haemorrhagic blood vessels (Hbv), atretic secondary follicle (Af), and a mass of corpus luteum; ovarian cortex with display of different levels of follicular developments with some eroding granulosa cells, ovarian stroma with papillary tumour appearance, atrophying primary and secondary follicles and haemorrhagic blood vessels. Graafian follicle (Gf), secondary follicle (Sf), tunica albuginea (Ta), stromal tumour (St), papillary tumour (Pt) ×100 magnification.

Histopathology of the testis showed abnormal histoarchitecture of the seminiferous tubule, mildly distorted orientation of the spermatogenic cells, displaced spermatids into the luminal space and areas of degeneration of the spermatids (Fig. 2). Also, the histopathology of the ovary showed some pathologies such as a cloggy appearing ovarian cortex with a display of a tumour-like cortical area, scanty display of primordial follicles, haemorrhagic blood vessels, atretic secondary follicle, eroding granulosa cells amongst others (Fig. 3).

## 4. Discussion

Evaluation and diagnosis for primary infertility in men often begin with semen analysis [17] especially as certain semen parameters are linked to the functional competence of the sperm cells [18]. Our findings showed that the herbal formulation caused a significant increase in the sperm count of the experimental rats. This may be attributed to the phytochemical constituents of the plant composition of the Dr Iguedo Goko Cleanser®. For instance, Onyejike et al. [19] reported that the herbal mixture contains tannins, saponins and flavonoids, and these plant chemicals are known to impact spermatogenesis. A typical example is tannins extracted from *Z. officinale* (a plant composition of the product) which have been reported to increase sperm parameters [20]. Despite the recorded significant increase in total sperm cell count of exposed male rats, the significant decrease in actively motile sperm cells, normal morphology as well as increase in non-motile sperm cells might indicate adverse exposure-associated effects of the polyherbal mixture. This could be considered systemic toxicity in male rats as earlier reported by Mylchreest et al. [21]. Under pathological conditions, free radicals are generated by endogenous or exogenous factors and/or a combination of both. As earlier reported by Kalender et al. [22], elevated reactive oxygen species (ROS) above normal limits induce lipid peroxidation in vital organs and systems, including the male reproductive system. Additionally, ROS can also set off a chain of events that leads to programmed cell death or apoptosis. Of course, ROS-induced DNA sperm damage can hasten the process of apoptosis, resulting in decreased sperm count [23]. As earlier reported by Jamalan et al. [24], metals are implicated in male infertility primarily by affecting the process of spermatogenesis vis-à-vis the quality of spermatozoa. There is a strong link between male infertility in metal-exposed human sperm samples and oxidative stress mediated by ROS generation [24]. Udom et al. [25] reported the presence of toxic heavy metals in the polyherbal mixture, and the reported spermatotoxic effects by our findings may probably be due to the repeated exposure of the male gonads to a mix of toxic metals. The histomorphological result of the testes revealed that the seminiferous tubules (STs) of the control male rats were good with normal histoarchitecture. Additionally, the spermatids, spermatogonia, and developing spermatozoa all displayed normal microarchitecture. There was no evidence of destructive or degenerative changes in the spermatogenic cell series, such as basal membrane breakdown of STs or degenerative changes in interstitial tissue (oedema or hyperaemia). Male rats exposed to high and low doses of the polyherbal mixture for 60 days revealed abnormal histoarchitecture, mildly distorted spermatogenic cells, displaced spermatids into the luminal space, and degeneration of the spermatids. Thus, this subchronic toxicity study showed that the herbal mixture may potentiate untoward changes in the seminiferous tubules, including structural changes in the spermatogenic cell series and interstitial tissue. Furthermore, decreased spermatogenesis explains the presence of spermatozoa with inordinate residual cytoplasm in the mid-piece, impacting negatively on the morphology of sperm cells. Reportedly, abnormal sperm morphology also occurs in the epididymis and seminiferous tubules [26]. Thus, the aforementioned condition could explain the observed changes in the morphology of the sperm cells in the experimental groups. Therefore, it could be said that the repeated or long-term use of the polyherbal mixture may induce structural and functional toxicities of the male gonads with consequential male infertility over time.

The histopathological assessment of the ovaries revealed some forms of pathologies such as a tumour-like cortical area with cystic space, scanty display of primordial follicles, haemorrhagic blood vessels, atretic secondary follicle mass of corpus luteum, eroding granulosa cells, ovarian stroma with papillary tumour appearance as well as atrophying primary and secondary follicles. The observed eroding granulosa cells indicate the occurrence of granulosa apoptosis, which is also considered the underlying mechanism of follicular atresia. Excessive cellular apoptosis is implicated in atrophy, and this might explain the atrophying of the primary and secondary follicles observed in the experimental female rats. The histopathological report on the ovaries is suggestive of a probable ovarian cyst and tumour in its early developmental stages. Again, it could be said that repeated and long-term exposure to the polyherbal mixture may induce structural toxicity of the ovary, with an attendant effect on reproductive functions.

Herbal drugs are usually regarded to be completely safe due to their natural sources, however, our findings reveal that Dr Iguedo Goko Cleanser® possesses the intrinsic capacity to induce an array of toxicities including oxidative stress, spermatotoxicity, structural toxicity of the gonads as well as hindered gonadal function. Therefore, extreme caution should be exercised during prolonged use of Dr Iguedo Goko Cleanser®, and wherever possible, be avoided. In Nigeria, and perhaps other countries in Sub-Saharan Africa, the concomitant use of conventional and herbal medicines are commonplace. Such practice is not without consequences especially as most patients who earlier presented to hospitals but absconded to patronize herbal remedies tend to return at a later stage with end-stage and multiple organ complications and in most cases, mortality ensues. The findings of the present study bring to the fore the toxic concerns of such unscrupulous practices. Our findings strongly advocate the need for toxicological screening of all purported herbal remedies and other quasi-drug formulations that are readily available in the Nigerian market.

## Figures and Tables

**Table 1:** Research design.

S/N	Group (n = 5)	Doses	Test Duration
1	C_1_	5 mL/kg W_d_	60 days
2	HF_1_	476.24 mg/kg GC	“
3	HF_2_	158.75 mg/kg GC	“
4	C2	5 mL/kg DW	“
5	HF3	476.24 mg/kg GC	“
6	HF4	158.75 mg/kg GC	“

W_d_ = Distilled water, GC = Goko Cleanser, C_1_ = control males, HF_1_ = high dosed males, HF_2_ = low dosed males, C_2_ = control females, HF_3_ = high dosed females, HF_4_ = low dosed females.
